# Fellowship Training After Four-Year Emergency Medicine Residency

**DOI:** 10.5811/westjem.63063

**Published:** 2026-05-19

**Authors:** Richard J. Hamilton, Lance B. Becker, Richard E. Wolfe

**Affiliations:** * Drexel University College of Medicine, Department of Emergency Medicine, Philadelphia, Pennsylvania; † North Shore University Hospital and Long Island Jewish Medical Center, Department of Emergency Medicine, Manhasset, New York; ‡ Donald and Barbara Zucker School of Medicine at Hofstra/Northwell, Institute of Bioelectronic Medicine, Uniondale, New York; § Feinstein Institutes for Medical Research, Department of Emergency Medicine, Manhasset, New York; || Harvard Medical School, Department of Emergency Medicine, Boston, Massachusetts; # Beth Israel Deaconess Medical Center, Department of Emergency Medicine, Boston, Massachusetts

To the Editor:

We thank Dr. Ehmann and colleagues for their thoughtful response and for the opportunity to clarify our interpretation of their work. We agree that their 2021 study does not demonstrate a statistically significant reduction in fellowship pursuit among graduates of their four-year program, and we appreciate their detailed description of updated outcomes from subsequent graduating classes. The disagreement is not about whether four-year programs can produce fellows, but whether mandating four years for all programs produces the same downstream effects. Our concern is not with the internal validity of their findings, but rather with their applicability to a proposed national mandate requiring that all emergency medicine (EM) residency programs adopt a four-year training format. A critical distinction exists between outcomes observed in a system where three- and four-year programs coexist and those that may reasonably be expected when choice is removed.

The overwhelming majority of EM training programs in our nation are currently three-year programs and are highly successful with no evidence of inferiority to four-year programs. Those trainees who enter four-year programs represent a smaller, self-selected cohort. They are, by inference, less constrained by time-to-practice, financial considerations, or opportunity cost, and may place greater intrinsic value on extended training, niche development, or academic productivity and, therefore, are not representative of the full applicant pool. In contrast, many EM applicants currently prioritize shorter training duration and have historically demonstrated a preference for three-year programs when given a choice. Under a mandated four-year model, the trainee population would necessarily include individuals for whom additional time in training carries meaningful personal, financial, or opportunity costs.

Accordingly, our original concern was not that four years of training intrinsically discourages fellowship pursuit, but rather that mandated extension of residency may alter downstream career decisions. For some trainees, the requirement to complete four years of residency could reasonably reduce willingness to pursue additional post-residency training, including fellowship, after already completing an extended training period.

These considerations are further amplified by the contemporary economic and practice environment facing EM trainees. Persistent inflation, erosion of physician purchasing power, and professional salaries that have not kept pace with rising costs increase the opportunity cost of prolonged training, particularly for trainees who enter residency training with substantial debt. At the same time, growing strains of EM practice—particularly crowding, boarding, and throughput pressures that disproportionately affect academic centers—may further influence career decision-making. Under these conditions, economic necessity and workforce pressures may increasingly compete with academic or fellowship aspirations, particularly when training duration is no longer elective.

We agree with Dr. Ehmann and colleagues that mentorship, subspecialty exposure, and structured academic development strongly influence career outcomes. However, these benefits reflect programmatic features rather than inherent consequences of training length. The experience of a single, highly resourced academic program with a niche-based curriculum and robust mentorship infrastructure—while informative—cannot be assumed to generalize to the broader national trainee population under a mandated four-year training requirement. Mandated expansion to a 4-year training length will not ensure mentorship, niche time, or scholarly infrastructure across all institutions.

At a time when EM faces declining applicant interest and increasing workforce uncertainty, preserving flexibility in training pathways may be particularly important. We believe that future discussions should focus on piloting evidence-based solutions to quantified educational gaps and identifying and disseminating effective curricular elements that promote academic development, rather than assuming that uniformly extending residency duration will yield similar outcomes across diverse programs and trainee populations.

This is an important topic for emergency medicine that deserves robust consideration and rational debate. There is no logical reason to make a hasty decision that lacks rudimentary data, raises issues of bias, and calls into question good decision-making processes. We appreciate the authors’ engagement in this important dialogue and welcome continued discussion regarding how best to support academic development, fellowship training, and workforce sustainability in emergency medicine.

